# A distal enhancer of GATA3 regulates Th2 differentiation and allergic inflammation

**DOI:** 10.1073/pnas.2320727121

**Published:** 2024-06-26

**Authors:** Takashi Kumagai, Arifumi Iwata, Hiroki Furuya, Kodai Kato, Atsushi Okabe, Yosuke Toda, Mizuki Kanai, Lisa Fujimura, Akemi Sakamoto, Takahiro Kageyama, Shigeru Tanaka, Akira Suto, Masahiko Hatano, Atsushi Kaneda, Hiroshi Nakajima

**Affiliations:** ^a^Department of Allergy and Clinical Immunology, Graduate School of Medicine, Chiba University, Chiba 260-8670, Japan; ^b^Department of Molecular Oncology, Graduate School of Medicine, Chiba University, Chiba 260-8670, Japan; ^c^Health and Disease Omics Center, Chiba University, Chiba 260-8670, Japan; ^d^Biomedical Research Center, Chiba University, Chiba 260-8670, Japan; ^e^Department of Biomedical Science, Graduate School of Medicine, Chiba University, Chiba 260-8670, Japan; ^f^Chiba University Synergy Institute for Futuristic Mucosal Vaccine Research and Development, Chiba 260-8670, Japan

**Keywords:** enhancer, GATA3, Th2 cell, ILC2, asthma

## Abstract

Asthma is a widespread airway disorder where GATA3-dependent Type-2 helper T (Th2) cells and group 2 innate lymphoid cells (ILC2s) play vital roles. Asthma-associated single nucleotide polymorphisms (SNPs) are enriched in a region located 926-970 kb downstream from GATA3 in the 10p14 (hG900). However, it is unknown how hG900 affects the pathogenesis of allergic airway inflammation. To investigate the roles of the asthma-associated GATA3 enhancer region in experimental allergic airway inflammation, we first examined the correlation between GATA3 expression and the activation of the hG900 region was analyzed by flow cytometry and ChIP-qPCR. We found that The activation of enhancers in the hG900 region was strongly correlated to the levels of GATA3 in human peripheral T cell subsets. We next generated mice lacking the mG900 region (mG900KO mice) were generated by the CRISPR-Cas9 system, and the development and function of helper T cells and ILCs in mG900KO mice were analyzed in steady-state conditions and allergic airway inflammation induced by papain or house dust mite (HDM). The deletion of the mG900 did not affect the development of lymphocytes in steady-state conditions or allergic airway inflammation induced by papain. However, mG900KO mice exhibited reduced allergic inflammation and Th2 differentiation in the HDM-induced allergic airway inflammation. The analysis of the chromatin conformation around *Gata3* by circular chromosome conformation capture coupled to high-throughput sequencing (4C-seq) revealed that the mG900 region interacted with the transcription start site of *Gata3* with an influencing chromatin conformation in Th2 cells. These findings indicate that the mG900 region plays a pivotal role in Th2 differentiation and thus enhances allergic airway inflammation.

## Significance

The activation status of the asthma-associated single nucleotide polymorphisms (SNPs) enriched region, which is located 900 kb downstream from GATA3 (hG900), and the levels of GATA3 expression were substantially correlated in human peripheral blood T cells. We generated mice with a deletion of the murine homologous region to hG900 (mG900KO mice). The mG900 region is critical in Type-2 helper T (Th2) cell differentiation but not pan-innate lymphoid cell (ILC) development, ILC2 development, and early T cell development in vivo. This is a report on mice lacking the critical enhancer of GATA3 specific for Th2 cell differentiation with minimal impact on ILC2 function and early T cell development.

Asthma is the most common allergic airway disease worldwide. Type-2 helper T (Th2) cells and group-2 innate lymphoid cells (ILC2s) play pivotal roles in asthma as the main producers of type-2 cytokines (1). Both Th2 cells and ILC2s depend significantly on a high amount of GATA3 (GATA-binding protein 3, encoded by the *GATA3* gene) for their development, maturation, and functions (2–5).

GATA3 is expressed in hematopoietic cells across various stages of their development. Recent studies involving enhancer deletions have illuminated the precise regulation of GATA3 expression in early T cell and ILC2 development (6). For instance, the deletion of 280 kb downstream region from *Gata3*-transcription start site (TSS) led to reduced GATA3 expression at double-negative (DN) 4 thymocytes, resulting in decreased CD4 single positive T cells in the thymus and spleen (7). The 280 kb deletion mice also showed decreased pan-ILCs (8). Meanwhile, deleting the 700 kb downstream region from *Gata3* significantly reduced mature ILC2s, with comparatively lesser effects on T cell development and Th2 differentiation (8).

Multiple genome-wide association studies (GWAS) have aimed to elucidate the underlying biology and predict susceptibility to asthma. The importance of single nucleotide polymorphisms (SNPs) within the 10p14 locus has been indicated in several independent studies, not only in asthma susceptibility but also in a broad spectrum of allergic diseases, including allergic rhinitis, atopic dermatitis, and eosinophilic granulomatosis with polyangiitis (9–14). A recent study revealed that *GATA3*-TSS interacts specifically with the SNP-accumulated region in memory Th2 cells (15), which suggests the causal involvement of the 10p14 locus in allergic diseases. However, the precise impact of this region on T cell development, Th2 differentiation, pan-ILC development, and ILC2 functions, especially during allergic inflammation, remains to be elucidated. In this study, we generated mice with a deletion of the homologous region to the SNP-enriched region. The mice lacking this region exhibited intact early T cell and ILC development in steady-state conditions and ILC2 function in papain-induced models yet demonstrated a lack of Th2 differentiation in house dust mite (HDM)-induced models. These findings unveil the pivotal role of the SNP-enriched region in Th2 differentiation.

## Results

### The Activation of the Human G900 Region Is Associated with GATA3 Expression in Th2 Cells.

**Fig. 1.**
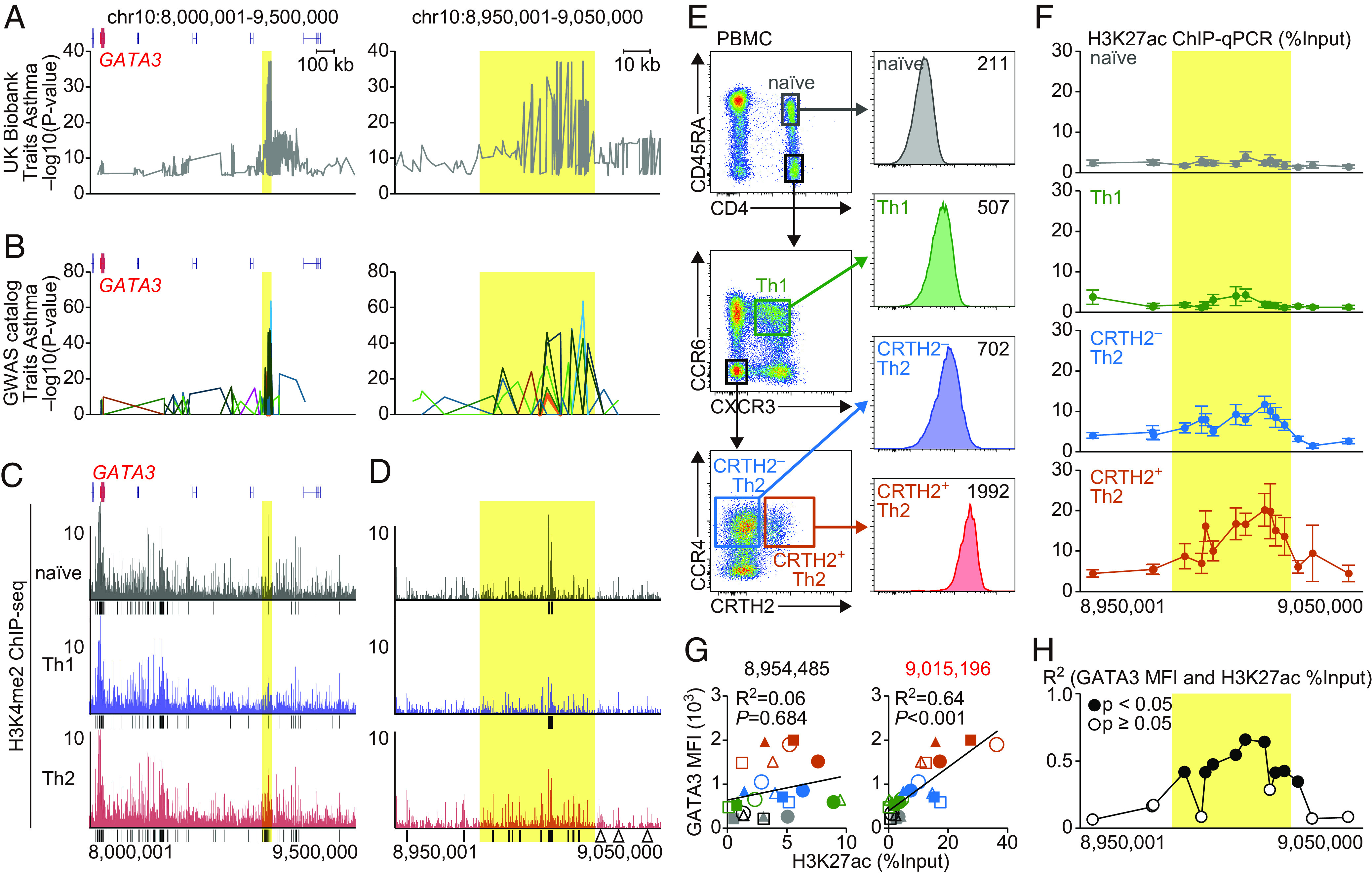
The activation of the human G900 region is associated with GATA3 expression in Th2 cells. (*A* and *B*) Asthma-associated SNPs of UK Biobank resource (*A*) and GWAS catalog (*B*). The yellow rectangle indicates the hG900 region. Line colors in (*B*) indicate the individual reports; see *SI Appendix*, Fig. S1. (*C* and *D*) UCSC tracks of H3K4me2 ChIP-seq analysis for the indicated T cell subsets (naïve CD4+ T cells, Th1 cells, and Th2 cells) derived from PBMCs of asthma patients (GSE53646) (19). The average of ChIP-seq counts was calculated from subjects 1, 5, 7, 8, and 9. Black bars indicate peaks, and the triangles indicate control regions. (*E*) GATA3 expression of naive CD4^+^ T cells, Th1 cells, CRTH2^−^ Th2 cells, and CRTH2^+^ Th2 cells in PBMCs harvested from healthy subjects. (*F*) Helper T cell subsets were sorted based on (*E*) and subjected to ChIP-qPCR. Line plots of %Input of H3K27ac levels on the peaks and control regions are shown as mean ± SE. (*G* and *H*) The correlation coefficient between H3K27ac levels (%Input) and GATA3 expression. (*G*) *Left*: the correlation at chr2:8,954,485 [black arrowhead in (*F*)]. *Right*: the correlation at chr2:9,015,196 [red arrowhead in (*F*)]. Dot shapes indicate each subject, and dot colors indicate the Th cell subsets in (*E*). Filled dots indicate male and open dots indicate female. Multiple comparisons were corrected by Holm’s method. (*H*) R^2^ at each peak. Empty circle: adjusted *P*-value ≥ 0.05, filled circle: adjusted *P*-value < 0.05. (*E*–*H*) Data are representative of six subjects in three independent experiments.

Several asthma-associated SNPs have been reported to be located in the region around the *Gata3* gene body (16–18). By analyzing asthma-associated SNPs from the UK Biobank and GWAS catalog, we confirmed that these SNPs were highly enriched in the region located 926 to 970 kb downstream from *GATA3* (referred to as the hG900 region) (Fig. 1 *A* and *B* and *SI Appendix*, Fig. S1 *A* and *B*).

To identify specific regulatory elements within the hG900 region in CD4^+^ T cells, we analyzed publicly available datasets of chromatin immunoprecipitation sequencing (ChIP-seq) of peripheral blood CD4^+^ T cell subsets obtained from individuals with asthma (19) (Fig. 1 *C* and *D*). H3K4me2 ChIP-seq data revealed the presence of eleven enhancers around the hG900 regions in CCR4^+^ Th2 cells (black bars in Fig. 1*D*). In contrast, only a few enhancers were identified in naive T cells and type-1 helper T (Th1) cells. These results suggest that the hG900 region contains clusters of Th2 cell-associated regulatory elements.

To further validate the relationship between these enhancers and GATA3 expression in T cells, we examined the levels of GATA3 and the enhancer activation in peripheral CD4^+^ T cell subsets from healthy subjects. GATA3 expression varied in a differentiation stage-dependent manner among CD4^+^ T cell subsets. Naive CD4^+^ T cells displayed low levels, while Th1 cells showed slight elevation (Fig. 1*E*). CRTH2^–^ Th2 cells exhibited a higher expression of GATA3, and notably, the highest expression of GATA3 was observed within CRTH2^+^ memory Th2 cells (Fig. 1*E*). Consistently, histone activation marks, H3K27ac, on the enhancers in the hG900 region were elevated in CRTH2^+^ Th2 cells (Fig. 1*F*). Furthermore, when comparing the expression of GATA3 and the activation status of enhancers, a positive correlation was observed between GATA3 levels and the activation of enhancers within the hG900 region, including chr10:9015196. Conversely, enhancers outside the hG900 region, such as chr10:8954485, showed no significant correlation (Fig. 1 *G* and *H*). These results suggest that GATA3 expression is up-regulated in Th2 cells through the enhancer activities in the hG900 region.

### The Murine Homologous Region to the hG900 Region Is Activated in Th2 Cells In Vivo and In Vitro.

To elucidate the precise role of the hG900 region, we aimed to investigate the functional loss of a murine counterpart. The murine homologous region corresponding to human 10p14 is located in murine chromosome 2 in the opposite direction, and the murine homologous region to the hG900 region is positioned 906 to 935 kb downstream from *Gata3*-TSS (referred to as mG900 region) (*SI Appendix*, Fig. S2 *A* and *B*). The mG900 region was activated in lung CD4^+^ T cells isolated from mice with HDM-induced allergic airway inflammation but not in lung CD4^+^ T cells from control mice (*SI Appendix*, Fig. S2 *A* and *B*). Consistently, cultured Th2 cells but not Th0 cells nor Th1 cells exhibited strong mG900 activation (*SI Appendix*, Fig. S2*C*) similar to the hG900 region in CRTH2^+^ Th2 cells (Fig. 1*F*). These findings imply functional similarity between the mG900 and hG900 regions. Consequently, we generated mice lacking the entire mG900 region (chr2:8936092-8980864) using the CRISPR-Cas9 system (mG900KO mice) (*SI Appendix*, Figs. S2*C* and S3).

### The mG900 Region Is Dispensable for Lymphocyte Development and ILC2 Function.

**Fig. 2.**
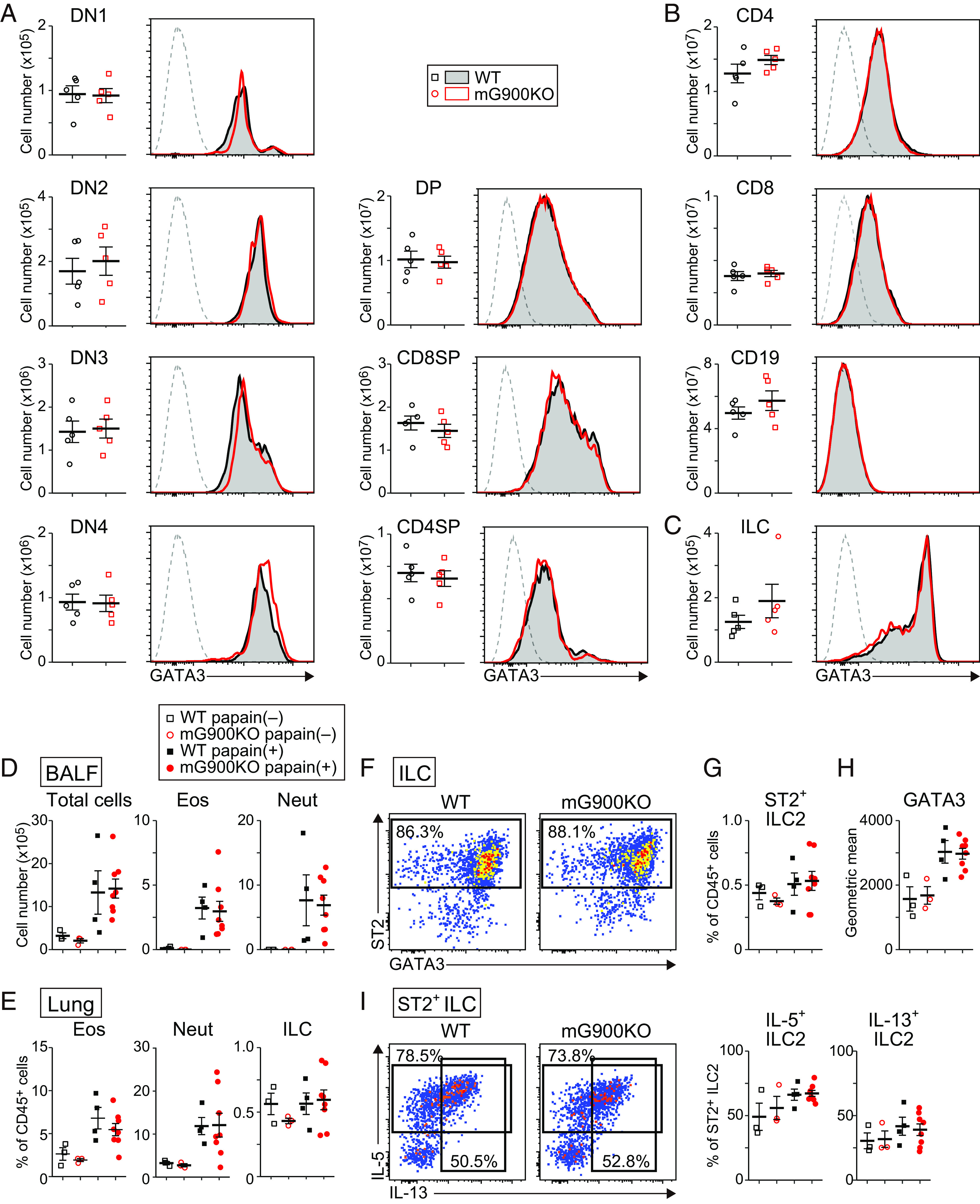
The mG900 region is dispensable for lymphocyte development and ILC2 function. (*A*–*C*) The numbers and GATA3 expression of thymocyte subpopulations (*A*), splenic lymphocytes (*B*), and lung ILC (*C*) in steady-state conditions. DN1: Lin1^–^Thy1^+^CD44^+^CD25^–^ cells, DN2: Lin1^–^Thy1^+^CD44^+^CD25^+^ cells, DN3: Lin1^–^Thy1^+^CD44^–^CD25^+^ cells, DN4: Lin1^–^Thy1^+^CD44^–^CD25^–^ cells, DP:CD4^+^CD8^+^ cells, CD8SP: CD4^–^CD8^+^ cells, CD4SP: CD4^+^ CD8^–^ cells. Lin1: CD3ε, CD4, CD8, TCRγδ, DX5. CD4: CD3ε^+^CD4^+^CD8^–^CD19^–^ cells, CD8: CD3ε^+^CD4^–^CD8^+^CD19^–^ cells, CD19: CD3ε^–^CD19^+^ cells. ILC: CD45^+^Lin2^–^CD90^+^ cells. Lin2: B220, CD3ε, CD4, CD5, CD8, CD11b, CD11c, CD19, Gr-1, NK1.1, Ter119. (*D*–*H*) mG900KO mice and littermate WT mice were subjected to papain-induced allergic airway inflammation as described in *SI Appendix*, Fig. S4*A*. The cell distribution of the BALF (*D*) and lung (*E*–*G*) and GATA3 levels of lung ST2^+^ILC2s (*H*) were shown. (*I*) Single-cell suspensions of the lung were stimulated with PMA/ionomycin in the presence of monensin for 3 h. Intracellular cytokine staining for IL-5 and IL-13 in ILC2 was performed. The data were representative of two independent experiments. n = 5, each genotype (*A*–*C*), and n = 3 for untreated mice, n = 4 for papain-induced WT mice, n = 8 for papain-induced mG900KO mice (*D*–*I*). Dot plots are shown as mean ± SE. Statistical analysis was performed with Welch’s *t* test.

Since GATA3 plays pivotal roles in the development and functions of hematopoietic cells, such as early T cells and ILCs, as well as Th2 differentiation (2, 3), we assessed whether T cell and ILC development are affected in mG900KO mice. The numbers of thymocytes, splenic lymphocytes, and lung ILCs were similar between mG900KO mice and littermate wild-type (WT) mice (Fig. 2 *A*–*C*). GATA3 expression in each population was also comparable between mG900KO mice and WT mice (Fig. 2 *A*–*C*). ILC2 differentiation from bone marrow ILC progenitors (ILCPs) in the in vitro culture was also similarly observed between mG900KO and WT ILCPs (20, 21) (*SI Appendix*, Fig. S5). These results imply that the mG900 region was dispensable for developing early T cells and ILCs and GATA3 expression in those cells in steady-state conditions.

A previous report indicates that the hG900 region is activated in peripheral blood ILC2s upon IL-25 and IL-33 stimulation (22). Thus, we assessed the role of the mG900 region in the in vivo functions of ILC2 using an ILC2-dependent papain-induced allergic airway inflammation (*SI Appendix*, Fig. S4*A*). The numbers of eosinophils and neutrophils in BALF, as well as the frequencies of eosinophils, neutrophils, and ILCs in the lung, were comparable between mG900KO mice and littermate WT mice (Fig. 2 *D* and *E*). Moreover, the number of ILC2s, the expression of GATA3 in ILC2s, and the production of IL-5 and IL-13 from ILC2s were similar between the two groups (Fig. 2 *F*–*I*). These results collectively indicate that the mG900 region is dispensable for GATA3 expression and functions in ILC2s in steady-state conditions and the papain-induced allergic airway inflammation.

### The mG900 Region Is Crucial for HDM-Induced Allergic Airway Inflammation and Th2 Differentiation In Vivo.

**Fig. 3.**
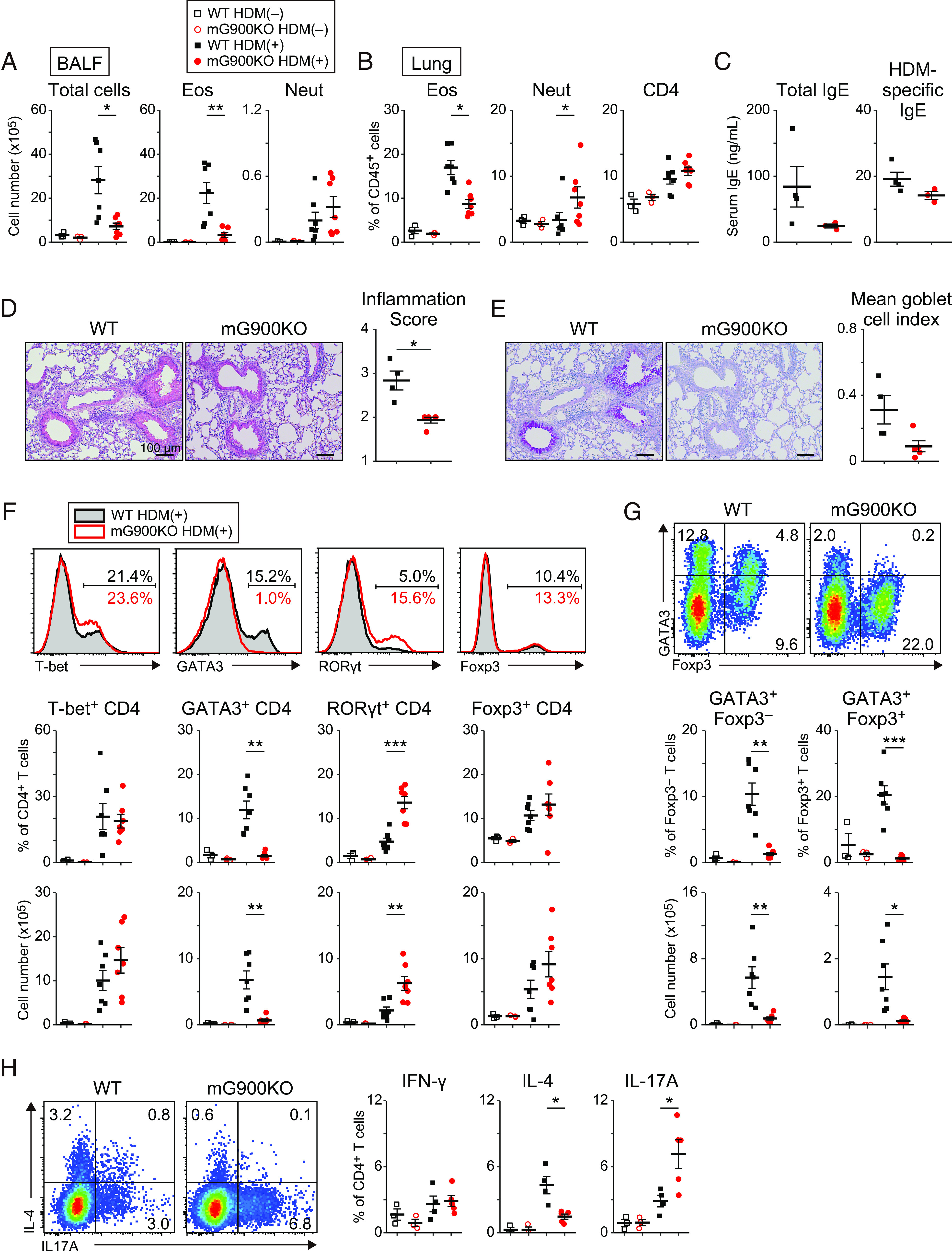
The mG900 region is crucial for HDM-induced allergic airway inflammation and Th2 differentiation in vivo. mG900KO mice and littermate WT mice were sensitized and challenged with HDM as described in *SI Appendix*, Fig. S4*B*. (*A* and *B*) Cell distribution in the BALF (*A*) and lung (*B*). The data for untreated mice were shared with Fig. 2 *D* and *E*. (*C*) Serum total IgE and HDM-specific IgE at day 13. (*D* and *E*) Hematoxylin–eosin (*D*) and PAS (*E*) staining of the left lower lobe and quantitative data. (*F* and *G*) Master transcription factor expression in lung CD4^+^ T cells. Red: mG900KO; black: littermate WT. (*H*) Single-cell suspensions of the lung were stimulated with PMA/ionomycin in the presence of monensin for 5 h. Representative intracellular IL-4 vs. IL-17A staining of lung CD4^+^ T cells and the frequencies of IFN-γ-, IL-4-, and IL-17A-producing cells are shown. (*A*, *B*, *F*, and *G*) The data were representative of three independent experiments, n = 7. (*C*) The data were representative of two independent experiments, n = 3 to 4. (*D*, *E*, and *H*) The data were representative of two independent experiments. n = 3 for untreated mice, n = 4 for HDM-induced WT mice, and n = 5 for HDM-induced mG900KO mice. (*A*–*H*) Dot plots are shown as mean ± SE. Statistical analysis was performed with Welch’s *t* test, *P < 0.05, **P < 0.01, and ***P < 0.001.

Next, we investigated the significance of the mG900 region in a T cell-dependent HDM-induced allergic airway inflammation (*SI Appendix*, Fig. S4*B*). mG900KO mice showed a noteworthy reduction in eosinophil infiltration in BALF and lung compared to WT mice (Fig. 3 *A* and *B*). Total IgE levels and HDM-specific IgE levels tended to decrease in mG900KO mice compared to WT mice (Fig. 3*C*). Lymphocyte infiltration around bronchi and blood vessels was attenuated in mG900KO mice (Fig. 3*D*), along with reduced mucin-producing goblet cells, which strictly depend on type-2 cytokines (23) (Fig. 3*E*). Importantly, GATA3 expression was significantly diminished in CD4^+^ T cells in mG900KO mice compared to those in WT mice, accompanied by upregulation of RORγt (Fig. 3*F*). The frequencies of T-bet-expressing cells and Foxp3-expressing cells were similar between mG900KO mice and WT mice. It has been shown that some Foxp3^+^ Treg cells also express GATA3 in allergic airway inflammation (24). Notably, Foxp3^+^ Treg cells expressing GATA3 were significantly decreased in mG900KO mice, indicating a shared mechanism for GATA3 expression in both Th2 cells and Treg cells during allergic airway inflammation (Fig. 3*G*). Consistent with the transcription factor profiles, IL-4-producing Th2 cells were significantly decreased in mG900KO mice, whereas IL-17A-producing Th17 cells were significantly increased (Fig. 3*H*), aligning with the increment of neutrophil infiltration in the lung of mG900KO mice (Fig. 3*B*).

We further investigated the impact of the mG900 region on the expression of master transcription factors in cultured CD4 T cells under helper T cell subset-polarizing conditions. Surprisingly, despite the activation of the mG900 region under Th2-polarizing conditions (*SI Appendix*, Fig. S2*C*), the expression of the master transcription factors, including GATA3 under Th2-polarizing conditions, was comparable between mG900KO mice and WT mice (*SI Appendix*, Fig. S6), presumably due to overpowering influence of in vitro IL-4 signaling. These results suggest that the mG900 region is indispensable and critical for Th2 differentiation in the HDM-induced allergic airway inflammation but not in the in vitro culture under Th2-polarizing conditions.

### The mG900 Region Contacts the *Gata3* Promoter and Enhancer in Th2 Cells.

**Fig. 4.**
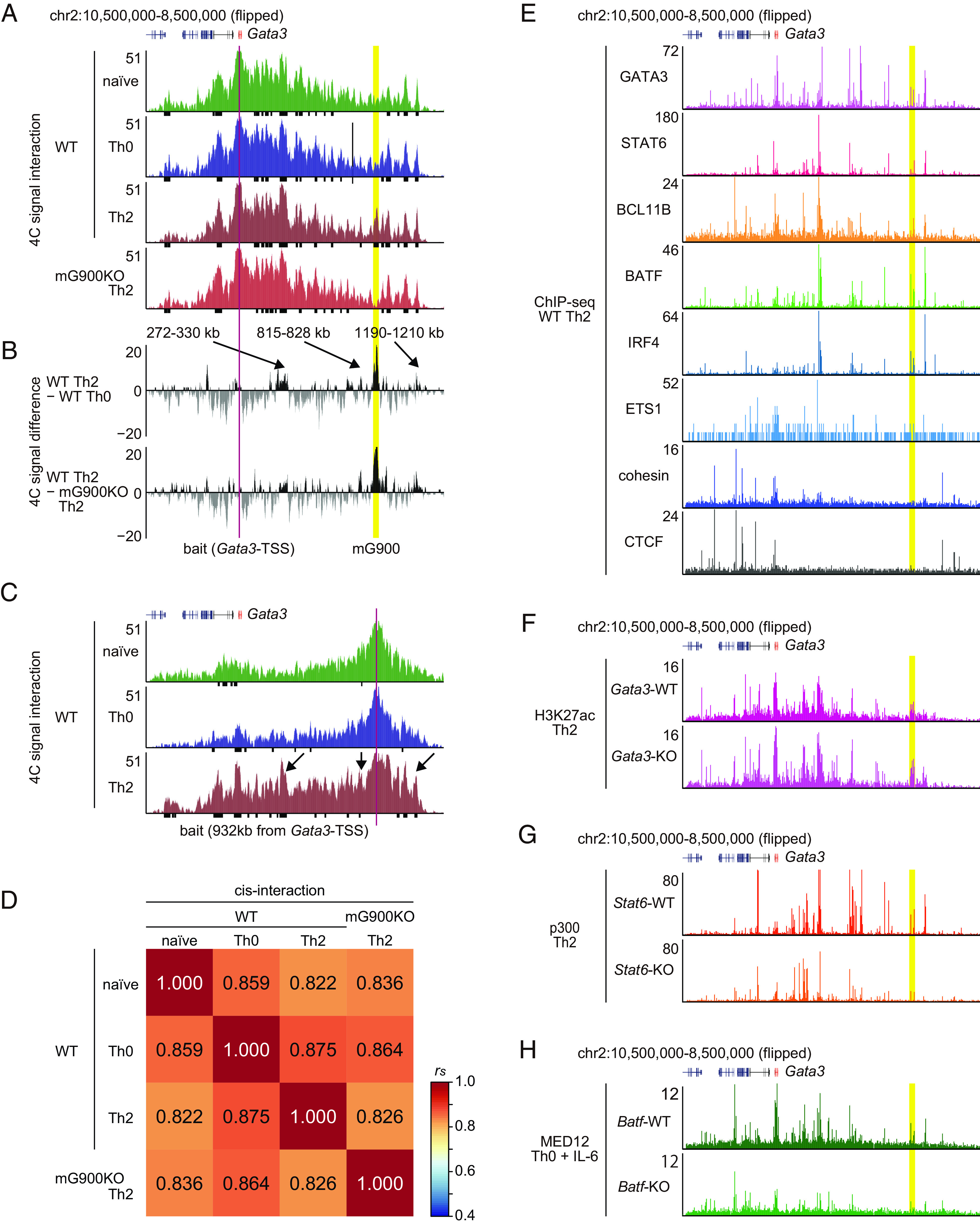
(*A*-*D*) The mG900 region contacts the GATA3 promoter and enhancers in Th2 cells. 4C-seq analysis for naive CD4^+^ T cells and cultured Th0/Th2 cells using the *Gata*3-TSS (*A* and *B*) and the region 932 kb downstream from *Gata3*-TSS, which is located in the mG900 region (*C*) as indicated baits. (*A* and *C*) UCSC tracks of 4C signal interaction. Black bars indicate significant interacting regions identified using w4Cseq. (*B*) Subtracted 4C-signal differences. (*D*) Similarity of 4C-signal interactions of (*A*) assessed by Spearman’s correlation. Cis-interaction: within chromosome 2. (*E*) UCSC tracks of ChIP-seq of GATA3 (GSE109109), STAT6 (GSE22104), BCL11B (GSE109109), BATF (GSE85172), IRF4 (GSE85172), ETS1 (GSE20898), cohesion (GSE66343), and CTCF(GSE66343) in cultured Th2 cells. (*F*) UCSC tracks of H3K27ac ChIP-seq of *Gata3*-WT and *Gata3*-KO Th2 cells (GSE237916). (*G*) UCSC tracks of p300 ChIP-seq of *Stat6*-WT and *Stat6*-KO Th2 cells (GSE40463). (*H*) UCSC tracks of MED12 ChIP-seq of *Batf*-WT and *Batf*-KO T cells under Th0 + IL-6 conditions (GSE123198). (*A*–*C*) Red rectangles indicate the bait position. (*A*–*C* and *E*–*H*) Yellow rectangles indicate the mG900 region.

A recent study proposes that memory Th2 cell identities coincide with the dynamic three-dimensional chromatin conformational changes, particularly around the *GATA3* locus (15). We investigated the significance of the mG900 region in the chromatin conformation surrounding *Gata3* in naive CD4^+^ T cells and in vitro differentiated Th0 cells and Th2 cells. Despite the mG900 region being distal from *Gata3*-TSS, the mG900 regions exhibited the most robust interaction with *Gata3*-TSS in Th2 cells compared to Th0 cells (Fig. 4 *A* and *B*). Additionally, in the significant interacting regions to *Gata3*-TSS in Th2 cells, three regions, located 272 to 330 kb, 815 to 828 kb, and 1,190 to 1,210 kb downstream from *Gata3*, displayed enhanced interaction with *Gata3*-TSS in Th2 cells compared to Th0 cells (Fig. 4*B*). Despite GATA3 expression remaining unaffected in vitro generated Th2 cells in mG900KO mice (*SI Appendix*, Fig. S6), intriguingly, Th2 cells of mG900KO mice exhibited reduced interaction in these regions (Fig. 4*B*). Consistently, the mG900 region exhibited a robust interaction to *Gata3*-TSS, along with those three regions, exclusively within Th2 cells (Fig. 4*C*). Furthermore, cis-interaction from *Gata3*-TSS in mG900KO Th2 cells displayed a stronger correlation with WT Th0 cells than with WT Th2 cells (Fig. 4*D*). These findings collectively indicate that the mG900 region promotes alterations in the chromatin conformation around *Gata3* to a form conducive to Th2 cells in vivo.

To explore the factor that activates the mG900 region, we reanalyzed deposit ChIP-seq data in Th2 cells. Among the factors we could investigate, GATA3, STAT6, BCL11B, BATF, and IRF4 bound to the mG900 region in cultured Th2 cells (25–27) (Fig. 4*E* and *SI Appendix*, Fig. S7*A*), suggesting that several key transcription factors for Th2 cells are involved in the activation of the mG900 region. Surprisingly, GATA3-deficient Th2 cells exhibited similar levels of H3K27ac signals on the mG900 region to WT Th2 cells (28) (Fig. 4*F* and *SI Appendix*, Fig. S7*B*). In contrast, STAT6-deficient Th2 cells exhibited reduced p300 bindings to the mG900 region (29) (Fig. 4*G* and *SI Appendix*, Fig. S7*C*). These findings suggest that the activation of the mG900 region partly depends on STAT6 but not on GATA3.

Recently, BATF has been reported to act as the chromatin conformation modulator, collaborating with ETS1 and CTCF (30). Although the mG900 region made a loop with *Gata3*-TSS in Th2 cells (Fig. 4 *A*–*C*) and BATF bound to the mG900 region (Fig. 4*E*), the bindings of ETS1, CTCF, and cohesion were not detected around the mG900 region in Th2 cells (31, 32) (Fig. 4*E* and *SI Appendix*, Fig. S7*A*). We thus evaluated the binding of MED12, another loop-generating molecule, in CD4 T cells cultured under Th0 + IL-6 conditions (30). BATF-deficient CD4 T cells exhibited reduced MED12 bindings on the mG900 region (Fig. 4*H* and *SI Appendix*, Fig. S7*D*), suggesting that BATF and MED12 may play a substantial role in the loop of *Gata3*-TSS and enhancers.

## Discussion

In this study, we have identified the pivotal role of the murine homologous region of human asthma-associated SNPs-enriched region in vivo differentiation of Th2 cells. Additionally, we have demonstrated that the mG900 region promotes interaction between *Gata3*-TSS and several enhancers in Th2 cells.

GWAS for various allergic diseases sheds light on the important factors underlying various allergic conditions. GATA3 is pivotal not only in allergic diseases but also in hematopoietic cell development and several malignancies. Numerous SNPs associated with allergic diseases, autoimmune diseases, and hematopoietic malignancies have been reported near the GATA3 locus. Intriguingly, SNPs associated with hematopoietic malignancies are enriched near the GATA3 gene body (18). In contrast, SNPs associated with allergic diseases are enriched within the hG900 region (18) (Fig. 1*A*). Immunoprecipitation using H3K4me2 to detect active and poised enhancers has suggested that enhancers within the hG900 region are formed specifically in Th2 cells (19). In our research, the detection of active chromatin states using H3K27ac has revealed a strong correlation between the activation state of 10 out of 11 enhancers within the hG900 region and GATA3 expression levels, even within Th2 cells. In murine systems, the mG900 region is also activated in Th2 cells and plays a critical role in Th2 differentiation under allergic inflammation. Collectively, these findings indicate the importance of the hG900 region in allergic diseases.

Recently, Chen et al. have explored functional sequences (FSs) within the entire 2 Mbp surrounding the GATA3 locus using tiling CRISPR screens in Jurkat cells (18). Notably, 100 bp deletion of seven enhancer regions around FS14, which is located at the center of the hG900 region, demonstrated no impact on GATA3 expression in human Th2 cells. Subsequent experiments involving the 100 bp deletion of known SNPs, such as rs12413578 and rs725861, revealed a mild decrease in GATA3 expression by the deletion of rs725861 but not rs12413578. However, the deleted region of rs725861 is not conserved in the mouse genome, making it impossible to analyze the role of rs725861 using the mouse system. In this study, we generated mG900KO mice to elucidate the role of SNP-enriched regions. We found that in vitro-generated Th2 cells can express normal levels of GATA3 in mG900KO mice, consistent with Chen’s findings from the enhancer deletion experiments around FS14. In contrast, in vivo induction of GATA3 expression and Th2 differentiation were severely impaired in mG900KO mice despite the lack of conservation of each human SNP in the murine genome. These findings suggest that GATA3 expression in Th2 cells in vivo may be attributed to the combined activity of multiple enhancers rather than individual SNPs.

Our results further underscore the cell- and stage-specific mechanisms governing GATA3 upregulation. GATA3 is essential at various stages in T cell development, including early T lineage progenitors, DN4, and CD4SP, and for the induction of Th2 cells (2, 7, 33, 34). During ILC development, GATA3 is necessary at two distinct stages: ILC progenitors and ST2^+^ ILC2 cells (3, 8). Recent analyses of enhancer-deletion mice have elucidated the cell- and stage-specific involvement of GATA3 enhancers in T cells and ILCs. Ohmura et al. identified murine TCE7.1 (280 to 287 kb downstream from *Gata3*-TSS) as the critical enhancer for early T cell development. TCE7.1-deficient mice exhibited a substantial reduction in thymic CD4 single-positive cells and peripheral naive CD4 T cells, while the number of CD44^+^CD62L^–^ memory T cells remained compatible between TCE7.1-deficient mice and WT mice (7). Similarly, Kasal et al. generated mice lacking the 280 to 287 kb region and demonstrated the critical role of 280 to 287 kb in pan-ILC development (8). Notably, residual CD4 T cells and ILC2s in TCE7.1- or 280 to 287 kb-deficient mice exhibit similar levels of GATA3 expression to those in WT mice (7, 8). Ohmura et al. also showed that TCE7.1 was inactivated in cultured Th2 cells compared to naive CD4 T cells using TCE7.1-reporter mice (7). These results suggest that TCE7.1 plays a fundamental role in early T cell and ILC development but not in Th2 cells or mature ILC2s. Meanwhile, the murine 672 to 762 kb region was identified as the ILC2-specific enhancer region (8). The 672/762 kb-deficient mice had fewer ILC2s and reduced GATA3 expression but exhibited minimal effect on other ILC subsets. Moreover, the 672/762 kb-deficient mice showed minimal impact on Th2 cell differentiation under HDM-induced allergic airway inflammation and no impact on ovalbumin (OVA)-induced allergic airway inflammation (8). Interestingly, mG900KO mice exhibited opposite phenotypes of the 672/762 kb-deficient mice. In mG900KO mice, T cell and ILC2 development seem intact in steady-state conditions, but in vivo Th2 differentiation was impaired. These three enhancer deletion mice revealed the complex regulation of GATA3 in the early T cell and ILC development, mature ILC2 development, and Th2 cell differentiation.

The mechanisms underlying the stage-specific GATA3 induction were investigated using the cytokine/transcription factor-deficient mice but still largely unknown except for the GATA3 induction in DN4 by Myb and in Th2 cells by IL-4/STAT6 signaling, IL-2/STAT5 signaling, and NOTCH signaling (2, 6). mG900KO Th2 cells have demonstrated reduced interactions between several enhancer regions and *Gata3*-TSS. While the interaction between the mG900 region and *Gata3*-TSS has been previously shown using Hi-C and promoter capture Hi-C (15), a decrease in other interactions due to the deletion of this region has not been reported. Recent research has revealed that the transcription factor BATF collaborates with ETS1 to recruit CTCF, facilitating the formation of chromatin loops and inducing changes in the chromatin structure of effector T cells (30). However, we found that the mG900 region did not show ETS1 and CTCF bindings in Th2 cells. Considering that the mG900 region contains multiple BATF binding sites and that BATF-deficient T cells showed reduced MED12 bindings on the BATF binding site, BATF may change chromatin structure in Th2 cells through the mG900 region. However, these observations are based on in vitro culture experiments. This notion should be investigated by further experiments in vivo.

This study used acute papain-induced models to access ILC2 function and HDM-induced models to evaluate Th2 cell differentiation. Recently, it has been reported that the role of ILC2s and Th2 cells is highly dependent on the models (35). Acute papain-induced models, the same models we used, depend on ILC2 but not Th2 cells, while chronic papain-induced models rely on both ILC2s and Th2 cells. In Th2 cell-dependent OVA-induced models, ILC2s are dispensable for eosinophilic inflammation but are stimulated and expanded by Th2 cell activation. Both acute and chronic papain-induced models are dependent on IL-33 but not IL-25 or TSLP, whereas OVA-induced models rely on TSLP but not IL-33 or IL-25 (35). Collectively, ILC2 and Th2 cells exhibit mutual effects, with the key molecules depending on the specific models employed. Considering mutual activation between ILC2 and Th2 cells, it is plausible that the deficiency of Th2 cells in mG900KO mice could impact ILC2 functions in other models. Further investigations using other experimental models are warranted to generalize the observed defect in Th2 cell differentiation and the intact function of ILC2s in mG900KO mice.

In conclusion, we unveiled the critical roles of the G900 region in Th2 differentiation in both humans and mice in vivo. The reasons for the different roles of the G900 region in Th2 cell differentiation in vivo and in vitro are currently unknown. Exploring the activation of the human and murine G900 region may be key to answering the fundamental question of how Th2 cells develop in vivo.

## Materials and Methods

### Ethics Statement and Healthy Volunteers.

The study was approved by the Ethics Committees of Chiba University (approval number: 3961) and was performed in accordance with the principles expressed in the Declaration of Helsinki. Informed consent was obtained from six Japanese healthy volunteers (three males and three females) aged between 28 and 44.

### Human Peripheral Blood Mononuclear Cell (PBMC) Preparation and Th Cell Subset Isolation.

PBMCs from healthy volunteers were isolated using Ficoll-Paque Premium (GE-Healthcare) and were stained with antibodies described below. We identified human naive T cells as CD4^+^ CD45RA^+^ cells and Th1 cells as CD4^+^ CD45RA^–^ CCR6^+^ CXCR3^+^ cells. Th2 cells, identified as CD4^+^ CD45RA^–^ CCR4^+^ CCR6^–^ CXCR3^–^ cells, were classified into two groups based on the expression of CRTH2.

### Mice.

C57BL/6 mice were purchased from CLEA Japan, Inc. mG900KO mice were generated as described below. Mice were housed in microisolator cages under specific pathogen-free conditions. Chiba University Animal Care and Use Committee approved the animal procedures used in this study.

### Generation of mG900KO Mice.

The gRNAs flanking the mG900 region were designed using the CHOPCHOP web tool (https://chopchop.cbu.uib.no/). Two crRNAs (GUAACCCAUACCUUAUACCCAGG and UAUUCAUGUAUUACGACCAGGGG) were annealed with tracrRNA (IDT) and bound with Cas9 protein (Invitrogen). Electroporation of ribonucleoprotein complexes was carried out on zygotes. After electroporation, eggs were cultured for 24 h, and two-cell stage embryos were transferred to the oviduct of pseudopregnant ICR females. The genotype of delivered mice was assessed with TOPO TA cloning (Thermo Fisher Scientific) and Sanger sequencing. Finally, two lines of mG900KO mice were generated, one with no indels and the other with a 1-bp deletion (*SI Appendix*, Fig. S3). All experiments were performed on both lines, and no significant differences were observed between the two lines.

### Antibodies and Flow Cytometry.

Antibody information is described in *SI Appendix*, Table S1. Cells were stained with a Zombie dye (BioLegend) and the indicated antibodies. For transcription factor analyses, cells were fixed and permeabilized with a Foxp3 Staining Buffer Set (Thermo Fisher Scientific) following the manufacturer’s instructions and were stained with antibodies for transcription factors. For intracellular cytokine analyses, cells were stimulated with Phorbol 12-myristate 13-acetate plus ionomycin in the presence of monensin (Sigma-Aldrich) for 3 or 5 h. Cells were fixed and permeabilized with a Fixation/Permeabilization Solution Kit (BD Biosciences) and stained with anticytokine antibodies. Cells were analyzed on a FACSCanto II (BD Biosciences). In some experiments, cell isolation was performed with an SH800 cell sorter (Sony Biotechnology) and BD FACSMelody (BD Biosciences).

### Cell Isolations.

Single-cell suspensions of the lungs were generated as previously described (36). Leukocytes were isolated from the interface between 40% and 80% Percoll gradient. Splenocytes and thymocytes were isolated by physical dissociation. All single cells were passed through a 30 µm filter before downstream experiments.

### Murine T Cell Culture.

Naive CD4^+^ T cells were isolated from lymph nodes and spleen using an EasySep Mouse Naive CD4^+^ T Cell Isolation Kit (STEMCELL Technologies) following the manufacturer’s instructions. Naive CD4^+^ T cells were stimulated with plate-bound anti-CD3ε antibody and soluble anti-CD28 antibody under helper T cell subset-polarizing conditions for 3 d (transcription factor analyses and 4C-seq) or 5 d (intracellular cytokine analyses). The conditions were as follows: Th0 conditions (no antibody, no cytokine), Th1 conditions [rmIL-12 (10 ng/mL, Peprotech), anti-IL-4], Th2 conditions [rmIL-4 (10 ng/mL, Peprotech), anti-IFN-γ], Th17 conditions [rmIL-6 (20 ng/mL, Peprotech), rmTGF-β (2 ng/mL, Peprotech), anti-IFN-γ, anti-IL-4], and Treg conditions [rmTGF-β, anti-IFN-γ, anti-IL-4].

### Murine Allergic Airway Inflammation.

Female mice were anesthetized with an intraperitoneal injection of midazolam, butorphanol, and medetomidine before treatment. For papain-induced airway inflammation, mice were intranasally administered 25 µg of papain (Nacarai Tesque) in 20 µL of PBS for three consecutive days. Twenty-four hours after the last challenge, BALF and lung cells were harvested (*SI Appendix*, Fig. S4*A*). For HDM-induced airway inflammation, mice were intratracheally administered 10 µg of HDM (Greer Laboratories) in 25 µL of PBS. Seven days later, mice were challenged with HDM (10 µg) for five consecutive days. Forty-eight hours after the last challenge, BALF and lung cells were harvested (*SI Appendix*, Fig. S4*B*).

### Histological Analysis.

Lung sections (3 µm thick) were stained with hematoxylin and eosin or periodic acid–Schiff (PAS) according to standard protocols. Inflammation scores and mean goblet cell index were calculated as previously described with minor modifications (37). Briefly, perivascular and peribronchial inflammation were scored as follows: 1 = single scattered leukocytes; 2 = aggregates less than 10 cells thick; 3 = aggregates about 10 cells thick; and 4 = numerous coalescing aggregates more than 10 cells thick. The total number of PAS-positive cells per total epithelial cell was evaluated for the goblet cell index. Scores were averaged from randomly selected three regions.

### ELISA.

The total IgE and HDM-specific IgE levels were determined using ELISA kits from Chondrex.

### ChIP-qPCR and ChIP-seq Analyses.

ChIP-qPCR and ChIP-seq analyses were performed as previously described with minor modifications (27). Briefly, cells were cross-linked with 1% formaldehyde at room temperature for 5 min before FACS sorting. Sorted cells were then sonicated for 12 cycles (30 s on, 30 s off) using a Bioruptor Pico (Diagenode). Sheared chromatin was incubated with anti-H3K27ac antibody (ab4729, Abcam) overnight and then immunoprecipitated by Dynabeads Protein A (Thermo Fisher Scientific). Immunoprecipitated chromatin–DNA complexes were washed seven times and were reverse cross-linked at 65 °C overnight. DNA fragments were harvested with phenol-chloroform extraction followed by ethanol precipitation. ChIP-qPCR was performed using specific primers on H3K4me2 peaks of human Th2 cells or conserved regions for murine T cell subsets (*SI Appendix*, Tables S2 and S3). Real-time PCR was carried out using Fast SYBR Green Master Mix and StepOnePlus (Applied Biosystems), and % input was calculated for each peak. ChIP-seq libraries were prepared with a ThruPLEX DNA-seq kit (Takara Bio), and sequencing was performed with an Illumina HiSeq 2500 in a 50 bp single-end mode.

### Data Integration and Correlation Analysis.

GATA3 expression of helper T cell subsets in six healthy volunteers was assessed using flow cytometry as described above. Percent input values of H3K27ac in the T cell subsets from the same volunteers were calculated as above. The data obtained from flow cytometry and ChIP-pPCR analysis were then merged. Pearson correlation coefficient between % Input values and GATA3 expression levels for each enhancer was calculated using R. Subsequently, *P*-values for 16 enhancers were adjusted using Holm’s method to account for multiple comparisons.

### 4C-seq Analysis.

4C-seq analyses were performed as previously described with minor modifications (38). Briefly, cells were cross-linked with 1% formaldehyde at room temperature for 10 min. Cells were lysed, and cross-linked DNA was digested with NlaIII (New England Biolabs) and then ligated using T4 DNA ligase (Thermo Fisher Scientific), followed by de-cross-link using Proteinase K (New England Biolabs). DNA fragments were subjected to a second digestion by BgIII (New England Biolabs) or DpnII (New England Biolabs) and circular ligation with T4 DNA ligase. Circular DNAs were subjected to PCR using inverse primers containing adaptors and indexes. For library PCR by the inverse primers designed at *Gata3*-TSS, the 4C template fragmented by NlaIII and BglII was used. For library PCR by the inverse primers designed at the region 932 kb downstream from *Gata3*-TSS, the 4C template fragmented by NlaIII and DpnII was used. The 4C-seq library was sequenced on a NovaSeq 6000 system (Illumina). Information on the design of 4C primers is shown in *SI Appendix*, Table S4.

### Computational Analysis.

Raw data of ChIP-seq for human CD4^+^ T cell subsets (GSE53646) (19) were reanalyzed. Five individuals (subjects 1, 5, 7, 8, and 9) were aligned to hg38 genome assembly using Bowtie1.2.0 (39). The aligned data of five individuals were merged and visualized using the make UCSC file program of Homer (40). The peaks of H3K4me2 were identified using the findPeaks program of Homer. H3K27ac ChIP-seq data for murine lung CD4^+^ T cells and transcription factor ChIP-seq for cultured murine CD4^+^ T cells (GSE20898, GSE22104, GSE40463, GSE66343, GSE85172, GSE109109, GSE123198, and GSE237916) (25–32) were aligned to mm10 genome assembly using Bowtie2 (41), and aligned data were visualized using Homer. 4C-seq data were analyzed and visualized using w4Cseq (42). Significant interacting regions were detected with the following parameters: size_inter: 400, size_intra: 50, window_intra:3000, and false discovery rate: 0.05. Interaction similarities were calculated using Spearman’s correlation to the 4C-signal on each bin.

Asthma-associated hSNP data were extracted from PhenoScanner (16, 43, 44) and GWAS catalog (17). Homologous blocks between hg38 - chr10: 7,000,001 - 10,000,000 and mm10-chr2: 7,800,001 to 10,800,000 were identified using Lastz with option “--chain”(45). The data of asthma-associated hSNP and conserved regions were visualized using R.

### Statistical Analysis.

Data are presented as means with SE. Welch’s *t* test was used to compare the two groups. *P* values < 0.05 were considered statistically significant.

## Supplementary Material

Appendix 01 (PDF)

## Data Availability

ChIP-seq and 4C-seq data are available from the Gene Expression Omnibus (GEO) database under accession numbers GSE218144 (46) and GSE241990 (47), respectively. Previously published data were used for this work (GSE53646 (19), GSE20898 (31), GSE22104 (26), GSE40463 (29), GSE66343 (32), GSE85172 (27), GSE109109 (25), GSE123198 (30), and GSE237916) (28).
