# Supplementary material for: A distal enhancer of GATA3 regulates Th2 differentiation and allergic inflammation
Source: Proc Natl Acad Sci U S A. 2024 Jun 26;121(27):e2320727121. doi: 10.1073/pnas.2320727121 (PMC11228505; doi:10.1073/pnas.2320727121)
Supplement: Supplementary file 1 — Appendix 01 (PDF) [file pnas.2320727121.sapp.pdf]

PNAS

Supporting Information for
Distal enhancer of GATA3 regulates Th2 differentiation and allergic inflammation

Takashi Kumagai, Arifumi Iwata*, Hiroki Furuya, Kodai Kato, Atsushi Okabe, Yosuke Toda, Mizuki Kanai, Lisa Fujimura, Akemi Sakamoto, Takahiro Kageyama, Shigeru Tanaka, Akira Suto, Masahiko Hatano, Atsushi Kaneda, and Hiroshi Nakajima*

*Corresponding author: Drs. Arifumi Iwata or Hiroshi Nakajima
Email: aiwata@chiba-u.jp (AI), nakajimh@faculty.chiba-u.jp (NH)

This PDF file includes:

Figures S1 to S7
Tables S1 to S4

Figure S1

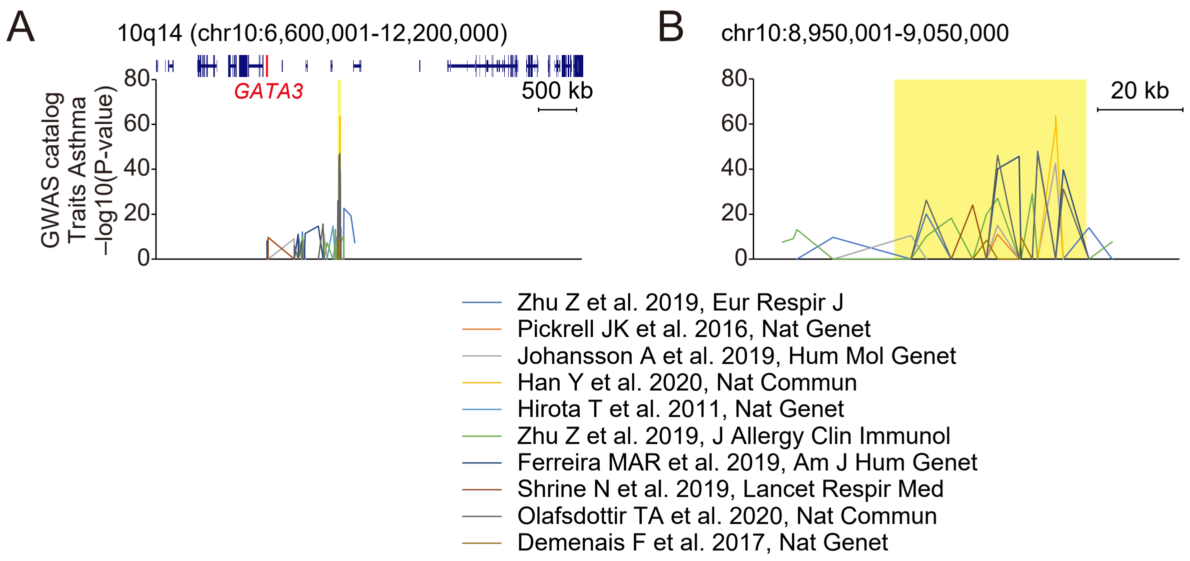

Figure S1. Nine hundred kilobase-downstream cis-regulatory element for human GATA3 is highly activated in Th2 cells.
(A, B) The line plot depicted the –log10(P-value) of asthma-associated SNPs at 10p14 (**A**) and around hG900 region (**B**). Data were collected from the GWAS catalog (https://www.ebi.ac.uk/gwas/). The yellow rectangles indicate hG900 region.

Figure S2

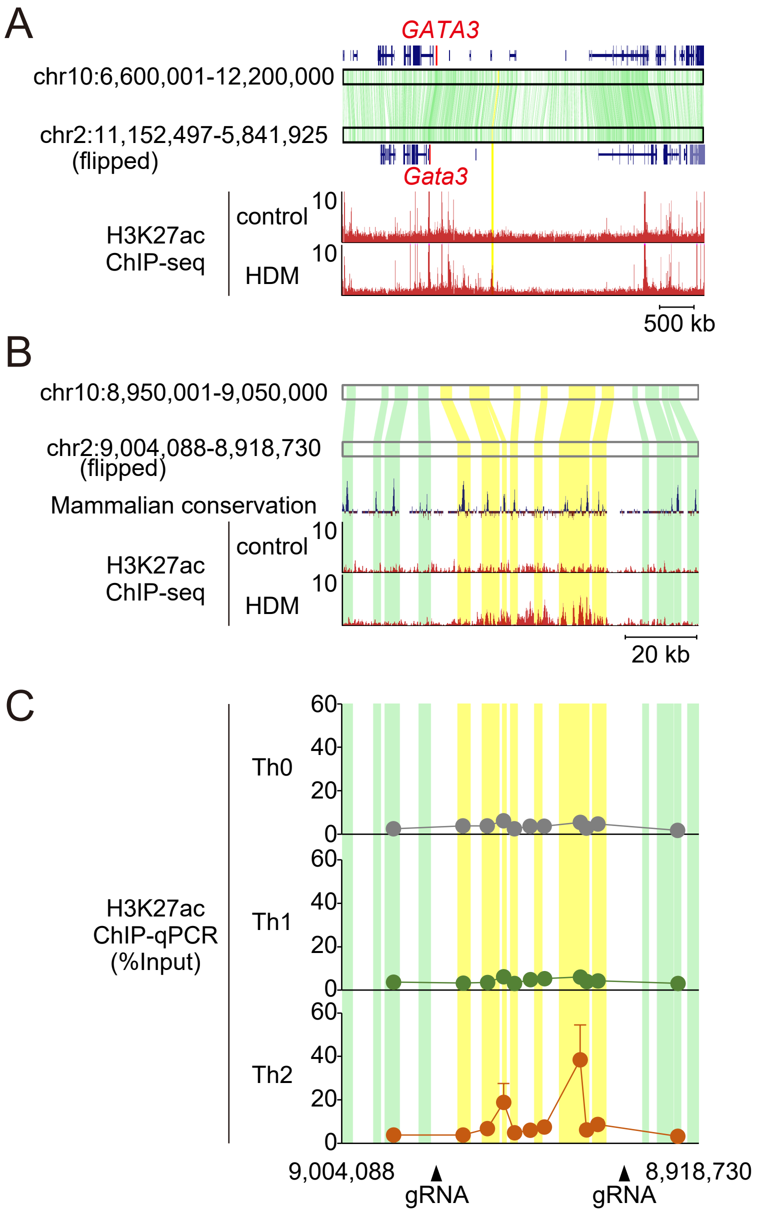

Figure S2. The murine homologous region to the hG900 region is activated in Th2 cells in vivo and in vitro.
WT mice were sensitized and challenged with HDM as described in Fig. S4B. **(A-B)** Upper tracks: Murine homologous region (outside hG900: green, inside: yellow) in 10p14 (**A**) and around hG900 region (**B**). Lower tracks: H3K27ac ChIP-seq data of CD4^{+} T cells from the lung of mice challenged with HDM and sensitized mice (control). (**C**) H3K27ac ChIP-qPCR of the homologous regions around mG900 region in cultured T cell subsets. Triangles indicate the cleavage sites for generating mG900KO mice by the CRISPR-Cas9 system.

Figure S3

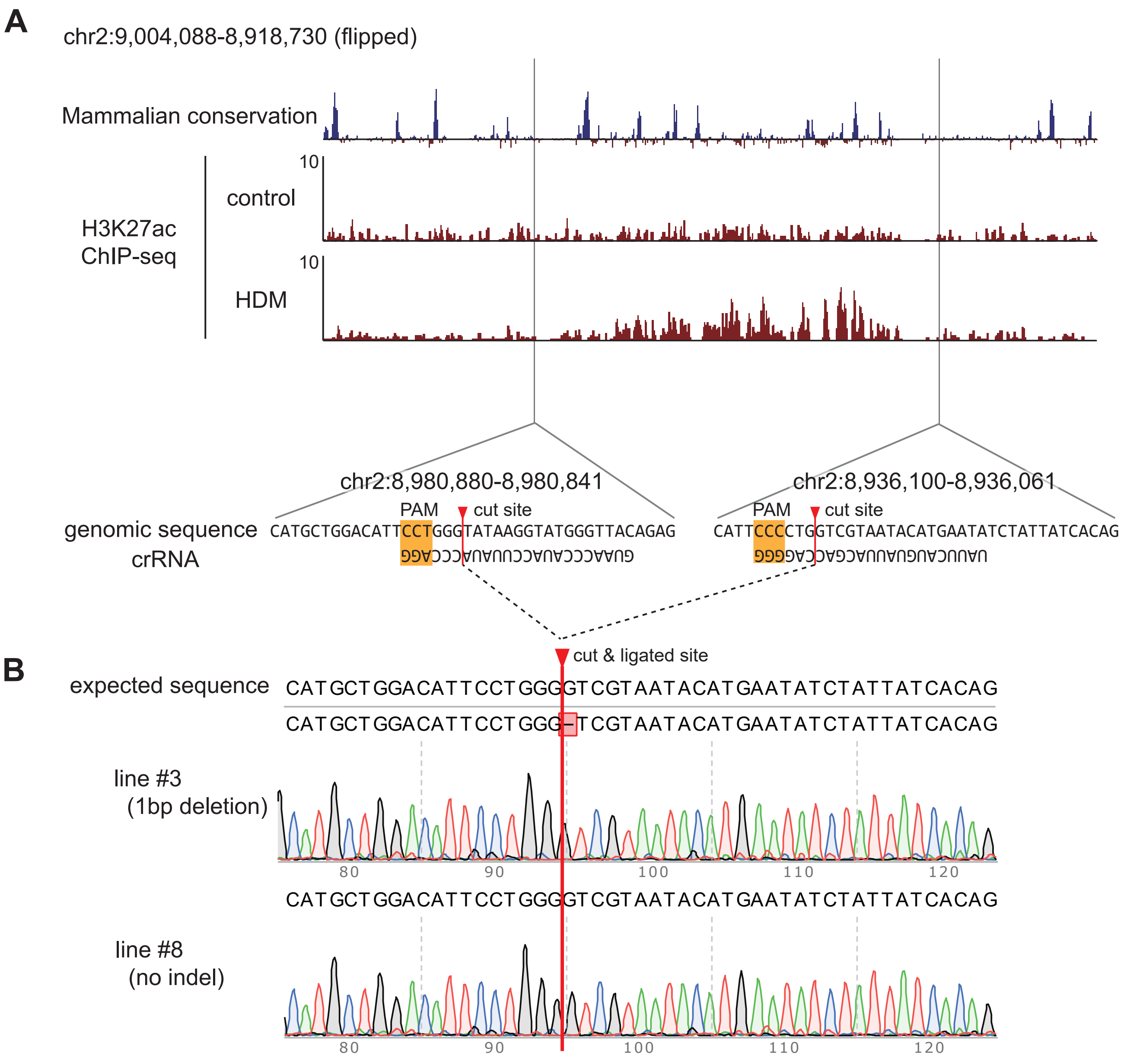

Figure S3. Generation of mG900KO mice.
(A) Upper panel: UCSC genome tracks around the mG900 region. Lower panel: Genomic sequence of upstream/downstream crRNA target regions. **(B)** DNA sequences of mG900 deleted loci from two lines of generated mG900KO mice.

Figure S4

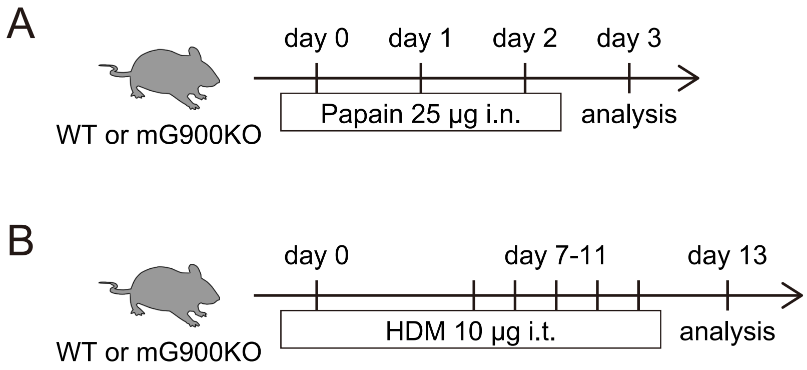

Figure S4. Experimental protocol of murine models of allergic airway inflammation. **(A)** Scheme of papain-induced airway inflammation. Twenty-five micrograms of papain were intranasally administered to mG900KO mice and control mice at day 0, day 1, and day 2. Twenty-four hours later, BALF and lung were harvested. **(B)** Scheme of HDM-induced airway inflammation. mG900KO mice and littermate wild-type (WT) mice were sensitized (day 0) and challenged (day 7-11) with 10 μg HDM intratracheally. Forty-eight hours later, BALF and lung were harvested. For control mice of Fig. S2, mice were sensitized with 10 μg HDM but not challenged with HDM.

Figure S5

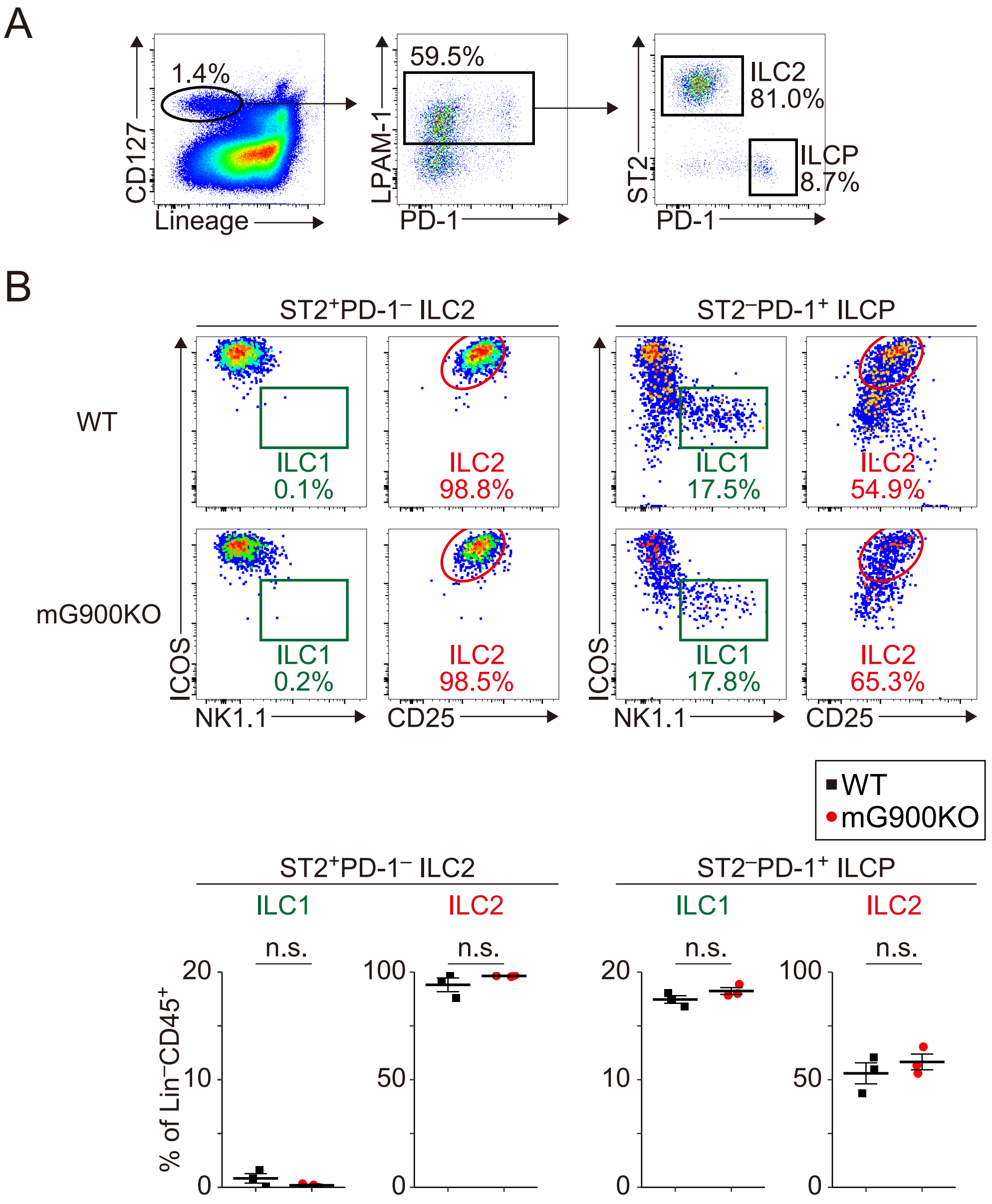

Figure S5. ILC2 development from ILC progenitor on OP9-DL1 cells.
Sorted ILC progenitors (ILCPs) and ILC2s from the bone marrow of WT mice and mG900KO mice were cultured on OP9-DL1 cells in the presence of IL-7 and SCF for 5 days. **(A)** Gating strategy for ST2^{+}PD-1^{-} ILC2 and ST2^{-}PD-1^{+} ILCP. **(B)** Frequencies of Lin^{-}CD45^{+}NK1.1^{+} ICOS^{int} ILC1s and Lin^{-}CD45^{+}ICOS^{hi}CD25^{+}PD-1^{-} cells. The plots are representative of three mice from two independent experiments.

Figure S6

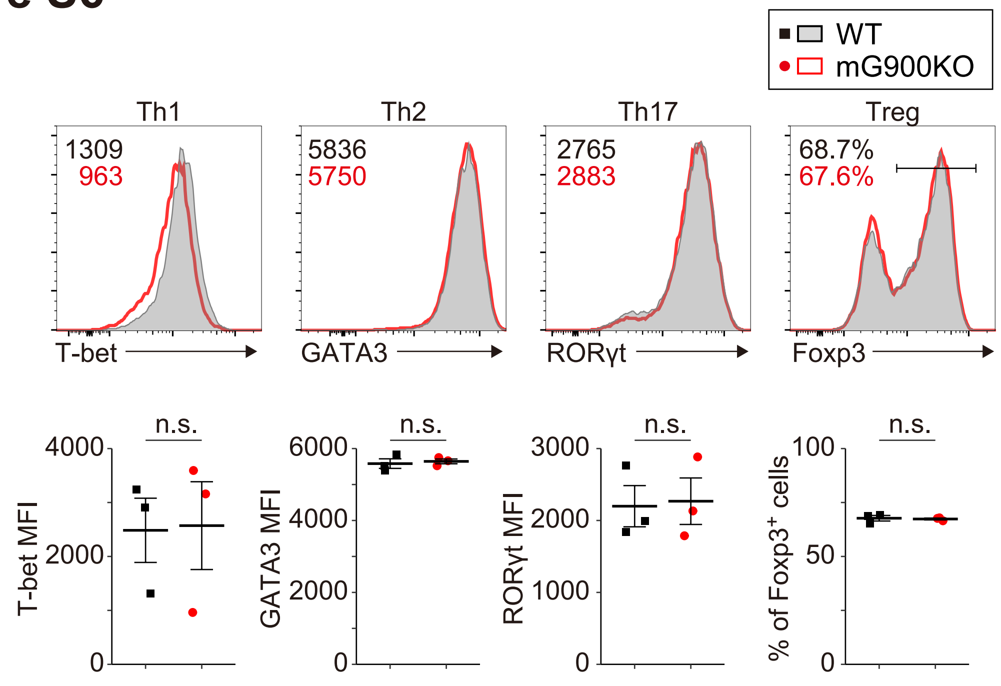

Figure S6. Master transcription factor expression in T cell subset-polarizing conditions. Naïve CD4 T cells were cultured under T cell subset-polarizing conditions for three days, and master transcription factor expression was analyzed. The plots are representative of three mice from two independent experiments.

Figure S7

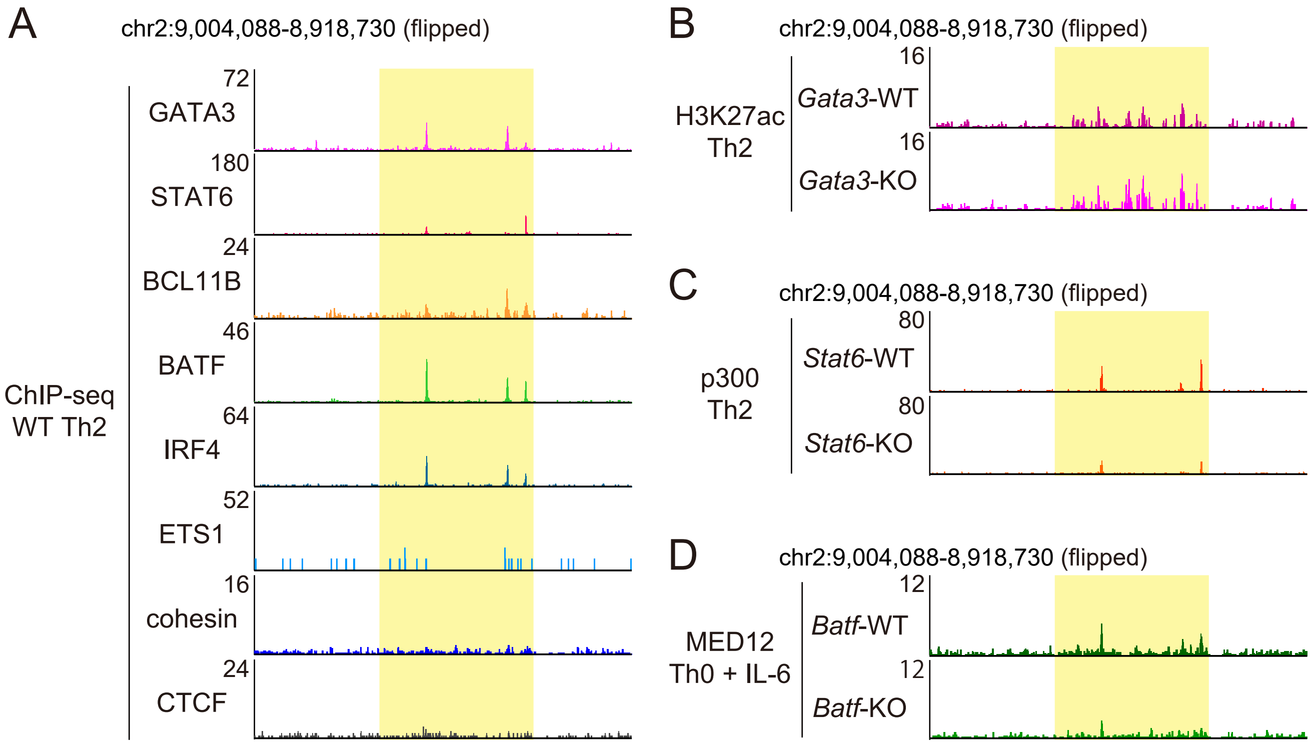

Figure S7. Transcription factor bindings on the mG900 region.
(A) UCSC tracks of ChIP-seq of GATA3 (GSE109109), STAT6 (GSE22104), BCL11B (GSE109109), BATF (GSE85172), IRF4 (GSE85172), ETS1 (GSE20898), cohesion (GSE66343), and CTCF (GSE66343) in cultured Th2 cells. **(B)** UCSC tracks of H3K27ac ChIP-seq of *Gata3*-WT and *Gata3*-KO Th2 cells (GSE237916). **(C)** UCSC tracks of p300 ChIP-seq of *Stat6*-WT and *Stat6*-KO Th2 cells (GSE40463). **(D)** UCSC tracks of MED12 ChIP-seq of *Batf*-WT and *Batf*-KO T cells under Th0 + IL-6 conditions (GSE123198). (**A-D**) Yellow rectangles indicate the mG900 region.

Table S1. The list of antibodies.

Antibodies	Company
Rat anti-mouse CD3ε FITC (Clone 145-2C11)	BioLegend
Rat anti-mouse CD3ε PE (Clone 145-2C11)	BD Bioscience
Rat anti-mouse CD3ε Brilliant Violet 510 (Clone 145-2C11)	BioLegend
Rat anti-mouse CD4 FITC (Clone RM4-5)	BioLegend
Rat anti-mouse CD4 PE (Clone RM4-5)	BioLegend
Rat anti-mouse CD4 PE/Cyanine7 (Clone GK1.5)	BioLegend
Rat anti-mouse CD4 Brilliant Violet 510 (Clone GK1.5)	BioLegend
Rat anti-human CD4 APC (Clone A161A1)	BioLegend
Mouse anti-human CD4 Brilliant Violet 510 (Clone SK3)	BD Bioscience
Rat anti-mouse CD4 Brilliant Violet 510 (Clone GK1.5)	BioLegend
Rat anti-Mouse CD8a FITC (Clone 53-6.7)	BD Bioscience
Rat anti-Mouse CD8a PE (Clone 53-6.7)	BD Bioscience
Rat anti-Mouse CD8a PE/Cyanine7 (Clone 53-6.7)	BioLegend
Rat anti-mouse/human CD11b FITC (Clone M1/70)	BioLegend
Rat anti-mouse/human CD11b PE (Clone M1/70)	BioLegend
Rat anti-mouse/human CD11b PE/Cyanine7 (Clone M1/70)	BioLegend
Hamster anti-mouse CD11c FITC (Clone N418)	BioLegend
Hamster anti-mouse CD11c PE (Clone N418)	BioLegend
Rat anti-mouse CD19 FITC (Clone 6D5)	BioLegend
Rat anti-mouse CD19 APC (Clone 1D3)	BD Bioscience
Rat anti-mouse CD44 PerCP/Cyanine5.5 (Clone IM7)	BD Bioscience
Rat anti-mouse/human CD45R/B220 FITC (Clone RA3-6B2)	BioLegend
Rat anti-mouse CD45 FITC (Clone 30-F11)	BioLegend
Rat anti-mouse CD45 PerCP/Cyanine5.5 (Clone 30-F11)	BioLegend
Mouse anti-human CD45RA PE/Cyanine7 (Clone HI100)	BioLegend
Rat anti-mouse CD49b FITC (Clone DX5)	BioLegend
Rat anti-mouse CD49b PE (Clone DX5)	BD Bioscience
Rat anti-mouse CD62L PE (Clone MEL-14)	Thermo Fisher Scientific
Rat anti-mouse CD90.2 PE/Cyanine7 (Clone 30-H12)	BioLegend
Rat anti-mouse CD90.2 Brilliant Violet 510 (Clone 53-2.1)	BioLegend
Mouse anti-human CD194 (CCR4) PerCP/Cyanine5.5 (Clone TG6/CCR4)	BioLegend
Mouse anti-human CD196 (CCR6) PE (Clone G034E3)	BioLegend
Mouse anti-human CD183 (CXCR3) PE (Clone G025H7)	BioLegend
Rat anti-human CD294 (CRTH2) Brilliant Violet 421 (Clone BM16)	BioLegend
Rat anti-mouse/human FOXP3 FITC (Clone FJK-16s)	Thermo Fisher Scientific
Rat anti-mouse/human FOXP3 PE (Clone FJK-16s)	Thermo Fisher Scientific
Mouse anti-mouse/human GATA-3 eFluor 660 (Clone TWAJ)	Thermo Fisher Scientific
Rat anti-mouse I-A/I-E FITC (Clone M5/114.15.2)	BioLegend
Rat anti-mouse Ly-6G/Ly-6C FITC (Clone RB6-8C5)	BioLegend
Rat anti-mouse Ly-6G APC (Clone 1A8)	BioLegend
Rat anti-mouse IFNγ Brilliant Violet 510 (Clone XMG1.2)	BioLegend
Rat anti-mouse IL-4 PE (Clone BVD4-1D11)	BD Bioscience
Rat anti-mouse/human IL-5 PE (Clone TRFK5)	BioLegend
Rat anti-mouse IL-13 eFluor 660 (Clone eBio13A)	Thermo Fisher Scientific
Rat anti-mouse IL-17 PerCP/Cyanine5.5 (TC11-18H10.1)	BioLegend
Rat anti-mouse IL-33Rα (IL1RL1, ST2) PE (Clone DIH9)	BioLegend

Rat anti-mouse IL-33Rα (IL1RL1, ST2) PE/Cyanine7 (Clone DIH9)	BioLegend
Rat anti-mouse IL-33Rα (IL1RL1, ST2) Brilliant Violet 421 (Clone DIH9)	BioLegend
Mouse anti-mouse NK-1.1 FITC (Clone PK136)	BioLegend
Mouse anti-mouse RORγt BV421 (Clone Q31-378)	BD Bioscience
Rat anti-mouse Siglec-F PE (Clone E50-2440)	BD Bioscience
Mouse anti-mouse/human T-bet PE (Clone 4B10)	Thermo Fisher Scientific
Hamster anti-mouse TCR β chain FITC (Clone H57-597)	BioLegend
Hamster anti-mouse TCR γ/δ FITC (Clone UC7-13D5)	BioLegend
Rat anti-mouse TER-119 FITC (Clone TER-119)	BioLegend
Ultra-LEAF™ Purified anti-mouse CD3ε Antibody (Clone 145-2C11)	BioLegend
Ultra-LEAF™ Purified anti-mouse CD28 Antibody (Clone 37.51)	BioLegend
Ultra-LEAF™ Purified anti-mouse IFN-γ Antibody (Clone XMG1.2)	BioLegend
Ultra-LEAF™ Purified anti-mouse IL-4 Antibody (Clone 11B11)	BioLegend
Anti-H3K27ac antibody (Clone ab4729)	abcam

Table S2. Human ChIP-qPCR primers.

Location	forward	reverse
8954485	GTGTGCACCCAAACTAGCC	CCAAACACAAACTGCATCCG
8975588	TCACAGCAAAATGAGTCGAAGG	TCTAGGGTAGTCTGTCTGTGG
8975988	GTGATAGTCAGTGTGGCATCTG	GTGTTAGTGGAGCCAGGAGA
8987000	ACCTTTGTCTGTAGTGAAATGCA	GGGAAATTGTTGTGTTGGCC
8992914	ATGCCTAGTATTCCCCAATGG	AGGAATCCTTGCTGTGATGAA
8994325	TTTGCTAATTCAGTGCTCACAG	TGATTTGACTGTGATTCCTCCA
8996985	CTTGTCTCCTCAGGGTTGGG	ATAACCTTGTGAGTGCTGCG
9005072	ACTTTGTCTCTAATGCATGGCC	ACATTACCTGCTGCATTCTCTG
9008487	TCTGGACCTTTAACTCTGCCA	CCCGACACAGCACTGAGA
9015196	CTCATATCCTGTGAAGTTTCCCA	GGACTTGGGAACTAACACTTGG
9017143	AGGGTTGACACTAATGCTTCA	TCCTGGAATAATCATTGGTCTGC
9019062	TCTATGGGAGATCTGTGGGC	AGTGGAAAGTGTCGATGTGG
9022237	GTGTGCACTTGGTTGGAGAG	CCATTTGGGGCATCATCTAGC
9026969	TGCAGGGTGTGTTCAGAAGA	CCTCCCATCCAGTGACTAGG

Table S3. Murine ChIP-qPCR primers.

Location	forward	reverse
887 kb	ATCCACTTATGGCTGGAGAGTC	TCCAGGCTATAGTTCTGTGTGC
904 kb	GCCTGTGTCTAGAGGGTAATGC	TCATGGAAACGGTGACTTATTC
909 kb	GAGACACTCAGTCGTGAAGTGG	ATACCACCAGGCAGTAGACAGG
913 kb	GCCTTTTAGCCCTCCCTTCT	TTCCAAATGGGTACCAAACC
916 kb	TTGGTAATACAGCATTTGGGTTC	CTCAGGGCAAAGAATGACTGA
920 kb	CATTCTGGTGGTGTCATTGG	AGCCCAGATGTTTTTCTTGG
923 kb	CAAACCCTCCTTCAGCTCAG	CTGTCTCTAGGGATCGCACTG
932 kb	TCAGGAGGACTCTGGACACC	AGTTAGGGCAGCCAGTTGAA
933 kb	TGGCAGGGAAGCTGTCTTAC	CCTCTCTGCTCCCAGTAAAGC
936 kb	CCGTACCTCCACAGAACACA	GAGGTAGGGGGAGGCATTTA
955 kb	CCTTTCAACAATCAAGTGGACA	GCCTGTGAACTGAAAACATTGA

Table S4. Murine 4C-seq primers.

Name	Sequence
Gata3-TSS-1st-F	TACACGACGCTCTTCCGATCTGGATACTGAGCTTCCATG
Gata3-TSS-1st-R	ACTGGAGTTCAGACGTGTGCTCTTCCGATCTAGAAGGTGGGAGGTTTTTC
932kb-1st-F	TACACGACGCTCTTCCGATCTACTAAGCTTTTACCCATG
932kb-1st-R	ACTGGAGTTCAGACGTGTGCTCTTCCGATCTAGTCTGGTGATTCAAAAGTA
4C-Universal-2ndPrimer-F	AATGATACGGCGACCACCGAGATCTACACTCTTTCCCTACACGACGCTCTTCCGATCT
4C-2ndPrimer-R-index7	CAAGCAGAAGACGGCATACGAGATGATCTGGTGACTGGAGTTCAGACGTGTGCT
4C-2ndPrimer-R-index8	CAAGCAGAAGACGGCATACGAGATTCAAGTGTGACTGGAGTTCAGACGTGTGCT
4C-2ndPrimer-R-index9	CAAGCAGAAGACGGCATACGAGATCTGATCGTGACTGGAGTTCAGACGTGTGCT
4C-2ndPrimer-R-index10	CAAGCAGAAGACGGCATACGAGATAAGCTAGTGACTGGAGTTCAGACGTGTGCT
4C-2ndPrimer-R-index11	CAAGCAGAAGACGGCATACGAGATGTAGCCGTGACTGGAGTTCAGACGTGTGCT
4C-2ndPrimer-R-index12	CAAGCAGAAGACGGCATACGAGATTACAAGGTGACTGGAGTTCAGACGTGTGCT
